# RETRACTION: Effect of Minoxidil Combined With Triamcinolone Acetonide on Alopecia Areata by Microneedle Injection

**DOI:** 10.1111/srt.70280

**Published:** 2025-10-21

**RETRACTION**: F. Wei, C. Cheng jun, S. Cheng, and L. Fang, “Effect of Minoxidil Combined With Triamcinolone Acetonide on Alopecia Areata by Microneedle Injection,” *Skin Research and Technology* 30, no. 4(2024):e13713, https://doi.org/10.1111/srt.13713.

The above article, published online on 17 April 2024 in Wiley Online Library (wileyonlinelibrary.com), has been retracted by agreement between *Skin Research and Technology*; and John Wiley & Sons Ltd. The article was accepted for publication following an insufficient peer review process that does not meet the standards of the journal's peer review policy. Furthermore, several scientific inconsistencies between data presented and reported results were identified, and the article lacks essential methodological details to interpret and reproduce the findings. Accordingly, the article has been retracted. The authors have been informed of the decision of retraction and disagree with it.

